# Behavioral responses of migratory caribou to semi-permeable roads in Arctic Alaska

**DOI:** 10.1038/s41598-025-10216-6

**Published:** 2025-07-09

**Authors:** Timothy J. Fullman, Kyle Joly, David D. Gustine, Matthew D. Cameron

**Affiliations:** 1 The Wilderness Society, Anchorage, Alaska 99501 USA; 2 Gates of the Arctic National Park and Preserve, Arctic Inventory and Monitoring Network, National Park Service, Fairbanks, Alaska 99709 USA; 3 Biological Resources Division, National Park Service, Anchorage, Alaska 99501 USA

**Keywords:** Barrier Behavior Analysis, Infrastructure, Migration, Movement, *Rangifer tarandus*., Animal migration, Behavioural ecology

## Abstract

Migration conveys many benefits to species, ecosystems, and people but relies upon connected landscapes. Anthropogenic development can present barriers for migrants, but many barriers are semi-permeable, allowing unhindered or delayed passage. We used a modified version of the Barrier Behavior Analysis (BaBA) to investigate seasonal movement responses to five roads in northwestern Alaska by adult female Western Arctic Herd caribou (*Rangifer tarandus*) from 2009 to 2024. Our analyses revealed some altered movement in response to all focal roads. We found that the roads were semi-permeable barriers to movement, with altered behaviors including bouncing away, moving back-and-forth, and tracing along roads. Overall, 63.1% of collared animals encountered (entered a road-specific buffer) at least one focal road. Of these, 61.5% displayed altered movements. At the scale of individual encounters with roads, we found altered movement in 27.1% of road encounters. Most encounters occurred during fall migration and caribou with altered behavior spent an average of 9.4 days longer near focal roads than those with unaltered movement. Altered movements were balanced among the behavioral responses. Most altered movements occurred near the Red Dog mining road (60.3%) or during fall migration (51.9%) but lasted longest during winter (17.2 days on average). We confirm prior findings of altered fall movements near the Red Dog road and demonstrate that movement behavior is also altered around other roads and in other seasons. Nonetheless, many collared caribou did not display altered movements in response to roads, emphasizing the need for further research to understand the mechanistic drivers of caribou movement responses. Given increasing pressures for infrastructure development and global challenges facing migratory species, it is critical to identify mitigation measures and inform management decisions seeking to balance responsible development with conservation of natural systems, including migratory species and the people that rely upon them.

**Supplementary Information:**

The online version contains supplementary material available at 10.1038/s41598-025-10216-6.

## Introduction

Migration, often conceived as back-and-forth movements between seasonal habitats, is a phenomenon shared among many animal taxa that conveys important benefits to individual fitness, ecosystem dynamics, and people. While migration is energetically costly for individuals^1–4^, animals that migrate can obtain access to increased resources, avoid challenging environmental conditions, reduce predation and disease risk, and increase offspring survival^5–11^. As animals migrate, they support ecosystem functioning by providing and redistributing nutrients, transporting other organisms, and altering trophic dynamics^12–15^. Additionally, migratory species often are relied upon by people, including many Indigenous groups that have strong cultural and subsistence connections with migrating animals^16–18^.

For migration to convey its myriad environmental and social benefits, animals need to be able to move between seasonal ranges. Habitat loss, fragmentation, or degradation, even in just a portion of the annual range, can have detrimental effects on migratory animals, hindering connectivity between habitats and reducing survival probability^19–21^. For terrestrial migrants, like many ungulates, one source of habitat loss and fragmentation is anthropogenic development, including linear barriers such as fences, railways, and roads, which can alter movement behavior and migratory connectivity^22–27^. Behavior may change in response to the physical movement obstructions presented by infrastructure or to the anthropogenic sounds, lights, movement, smells, and other stimuli associated with infrastructure^28–31^. Some anthropogenic features may serve as impermeable barriers, which preclude movement to portions of a species’ range and increase habitat fragmentation^32^. However, many types of development do not provide an absolute barrier to migratory movements. These semi-permeable barriers allow some or all individuals to pass unhindered or after a delay^32–34^. Landscape features may serve as barriers for some species while facilitating movement of other species^35–37^, leading to suggestions of viewing landscape features on a corridor–barrier continuum^38^. These different possible outcomes make it crucial to investigate how species respond to particular types of barriers in different contexts^32,39^ and may suggest opportunities for mitigation of barrier effects^40,41^. Improving our understanding of attributes of semi-permeable barriers and how animals respond to them is a critical step in balancing human development and wildlife connectivity.

Caribou, and conspecific reindeer (*Rangifer tarandus*), are the most abundant large terrestrial herbivore in the circumpolar Arctic^42–44^, with populations stretching across North America, Europe, and Asia^45,46^. Barren-ground caribou (*R. t. granti*), like those that live in northern Alaska, are renowned for their long-distance migrations, covering hundreds to thousands of kilometers each year in some of the longest overland migrations in the world^47,48^. This mobility allows caribou to move between different regions seasonally to take advantage of resources that change over space and time^49^, such as moving to areas with greater winter food availability and shelter and then returning to calving grounds^50–52^. Such movements also bring caribou into contact with many Indigenous people across the circumpolar Arctic, for whom caribou contribute to cultural well-being and food security^17,53–55^. Subsistence hunting of caribou provides an important source of nutrition, maintains cultural and spiritual connections, and supports customary and traditional ways of life^17,56,57^.

Although widely distributed, many caribou and wild reindeer populations have experienced declines over recent decades: more than 50% of migratory caribou and reindeer populations globally have declined over the past 20 years^58–61^. While caribou herds naturally fluctuate in size^62,63^, several herds show no sign of recovery after drastic declines of more than 90%, and some are at record low levels since written monitoring began^60,61^. In northwestern Alaska, the Western Arctic Herd (WAH) was the largest herd in Alaska, but has decreased 69% over the last two decades from a population high of 490,000 individuals in 2003^64^ down to 152,000 in 2023^65^. The marked decline in herd size led to restrictions on harvest^66^ and reinforced the importance of understanding factors that influence caribou populations. While numerous studies have documented responses of Alaskan caribou to roads and other infrastructure during the calving, post-calving, and insect harassment seasons^31,67–70^, less is known about caribou reactions to infrastructure at other times of the year, including the degree to which barriers are permeable to movement.

In this study, we employed a recently developed approach for identifying individual responses to potential barriers, the Barrier Behavior Analysis^34^ (BaBA), to investigate movement responses and permeability of roads in northwestern Alaska for barren-ground WAH caribou. Wilson et al.^33^ reported altered movement behavior of the WAH in proximity to a mining road in northwestern Alaska from 2009 to 2013. We expand upon this finding by investigating caribou movement responses to multiple roads across seasons in northwestern Alaska with 11 additional years of caribou location data. We predicted that movement behavior would be altered for some caribou in proximity to roads and that these alterations would affect time spent near roads. We also expected that multiple movement responses to roads would be detectable, including some caribou showing little or no alteration of movement behavior.

## Methods

### Study area

**Fig. 1:**
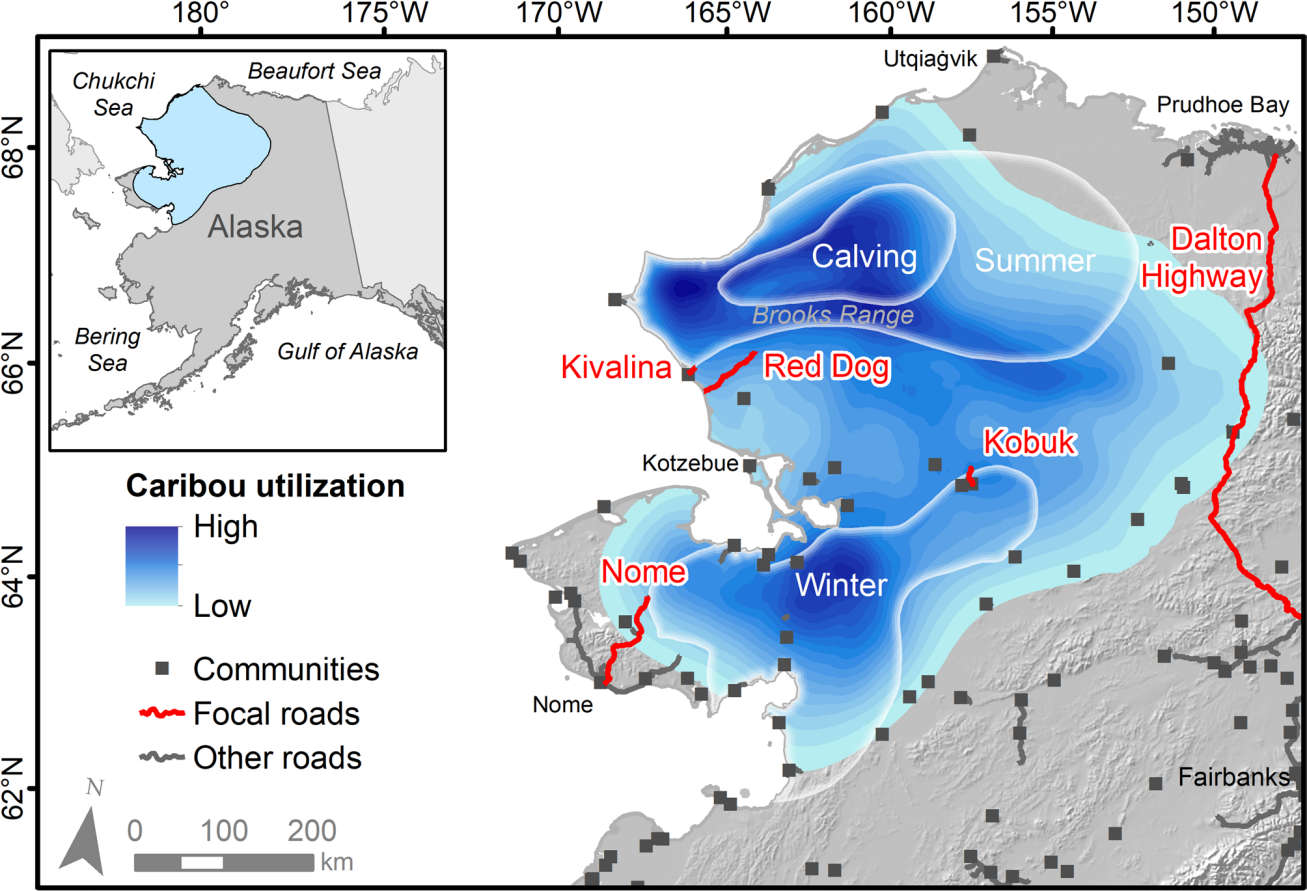
Seasonal ranges of the Western Arctic Herd (WAH) in northwestern Alaska and focal roads analyzed in this study (highlighted in red) from 2009–2024. Annual utilization intensity for WAH caribou is shown in blue and generalized seasonal range areas by white polygons. Note that there is considerable interannual variability in specific areas of use, especially during winter. Utilization intensity data and seasonal range polygons provided by the Alaska Department of Fish and Game.

The WAH moves among various seasonal ranges covering approximately 320,000 km^2^ in northwestern Alaska (Fig. 1). Generally, the herd calves in the foothills of the Brooks Range mountains in early June, spreads across the Brooks Range seeking relief from biting insects and foraging opportunities in July and August, and then migrates south towards their winter range^64,71^ (Fig. 1), though recent changes in the predominant winter range have been noted, with more animals staying further north or east of historic core winter ranges^71,72^. The herd is relied upon for subsistence harvest by Alaska Native and other hunters from nearly 40 communities in the range of the herd, as well as for hunting and tourism by those from different parts of Alaska and outside the state.

The majority of human habitation in the WAH range is characterized by small, isolated communities unconnected by a road system (Fig. 1). Access to most communities and other regions in the study area occurs by air or boat, with some overland travel via snow machines or vehicles on snow and ice roads during the winter^73–75^. Where roads do exist, their impacts on caribou and subsistence harvest have been of concern to some Alaska Native community members and other Alaskans^76–78^.

We analyzed five focal roads within the WAH range (Fig. 1). These were selected because they were regularly encountered by the WAH, covered different geographic regions within the herd’s range, and spanned an array of intensities of use. The DeLong Mountain Transportation System, commonly referred to as the “Red Dog road,” is an approximately 82 km gravel road providing access between the Red Dog Mine – a large zinc and lead mine opened in 1989 – and port facilities on the Chukchi Sea coast. Approximately 26 km to the north of this is a 12 km road providing the village of Kivalina with an evacuation route away from the coast (“Kivalina road”). This emergency access road was officially opened in 2020 following multiple years of planning and construction^79^ and since 2023 has provided access to a new school at the end of the road. However, community members from the village of Kivalina reported that the route was consistently used as an all-terrain vehicle (ATV) trail for hunting prior to establishment of a permanent road (D.A. Hansen, unpublished data). While the intensity of use and degree of environmental modification varied over time, we noted reactions to the route prior to 2020 and have retained it for the full period of our analysis. Further to the south, multiple roads originate from Nome, with the northernmost 67 km stretch intersecting the WAH winter range (“Nome road,” built around 1960). Along the eastern edge of the WAH range the Dalton Highway extends from the city of Fairbanks north to the Prudhoe Bay oil complex on the coast of the Beaufort Sea (“Dalton Highway,” built 1974, 163 km lie within the WAH annual range). Finally, the small Dahl Creek airstrip access and Bornite mining exploration roads are collectively referred to as the “Kobuk road” (approximately 24 km, built in the 1960s with exploration expanding since 2004). While currently isolated, the Bornite mine could be accessed by the proposed Ambler mining road^80^. Together, these five roads cover about 0.002% of the WAH’s annual range, assuming an average road footprint width of 18.9 m^81^. In addition to these five, there are smaller roads and ATV trails near communities in the range of the herd that were not included in our analysis.

### Telemetry data

To investigate movement responses of WAH caribou to the focal roads, we used caribou location data from GPS-collared adult females. Most collaring of Alaskan caribou focuses on females due to their critical contribution to population dynamics and the use of female calving distribution to define herd identity^82,83^. Collars were deployed by hand capturing via boat and helicopter-based net gunning by the Alaska Department of Fish and Game and National Park Service between 2009 and 2024. Animal handling was approved by the State of Alaska’s Animal Care and Use Committee protocol #2012-031R, 0040-2017-40, and 0040-2019-23 and was carried out in alignment with all requirements and guidelines. Most collars featured an 8-h fix interval, but some had a shorter interval, and others were variable over time. We standardized all data to an approximately 8-h fix interval, retaining the closest record within 2.25 h of the target 8-h times, constrained to a maximum of three fixes per day. Three animals had longer fix intervals of 24- or 120-h fixes that could not be matched to an approximately 8-h schedule and were excluded from further analysis. Animals with less than 4 months of data (corresponding to our longest season) were excluded to ensure sufficient sample size to conduct analyses.

### Movement classification using the BaBA

We characterized caribou movement responses to the focal roads using a modified version of the Barrier Behavior Analysis (BaBA) approach developed by Xu et al.^34^. In short, the BaBA compares movement parameters near a potential barrier with those away from barriers to identify altered movement behavior^34^. Preliminary testing of the default BaBA methods revealed poor alignment with caribou behavior. This was likely influenced by the much larger scale of movements exhibited by WAH caribou compared to the focal species of Xu et al.^34^ and the coarser telemetry fix interval in our study. As a result, we made extensive modifications to the BaBA approach, which are outlined below and more fully described in Supplementary Information 1.

**Fig. 2:**
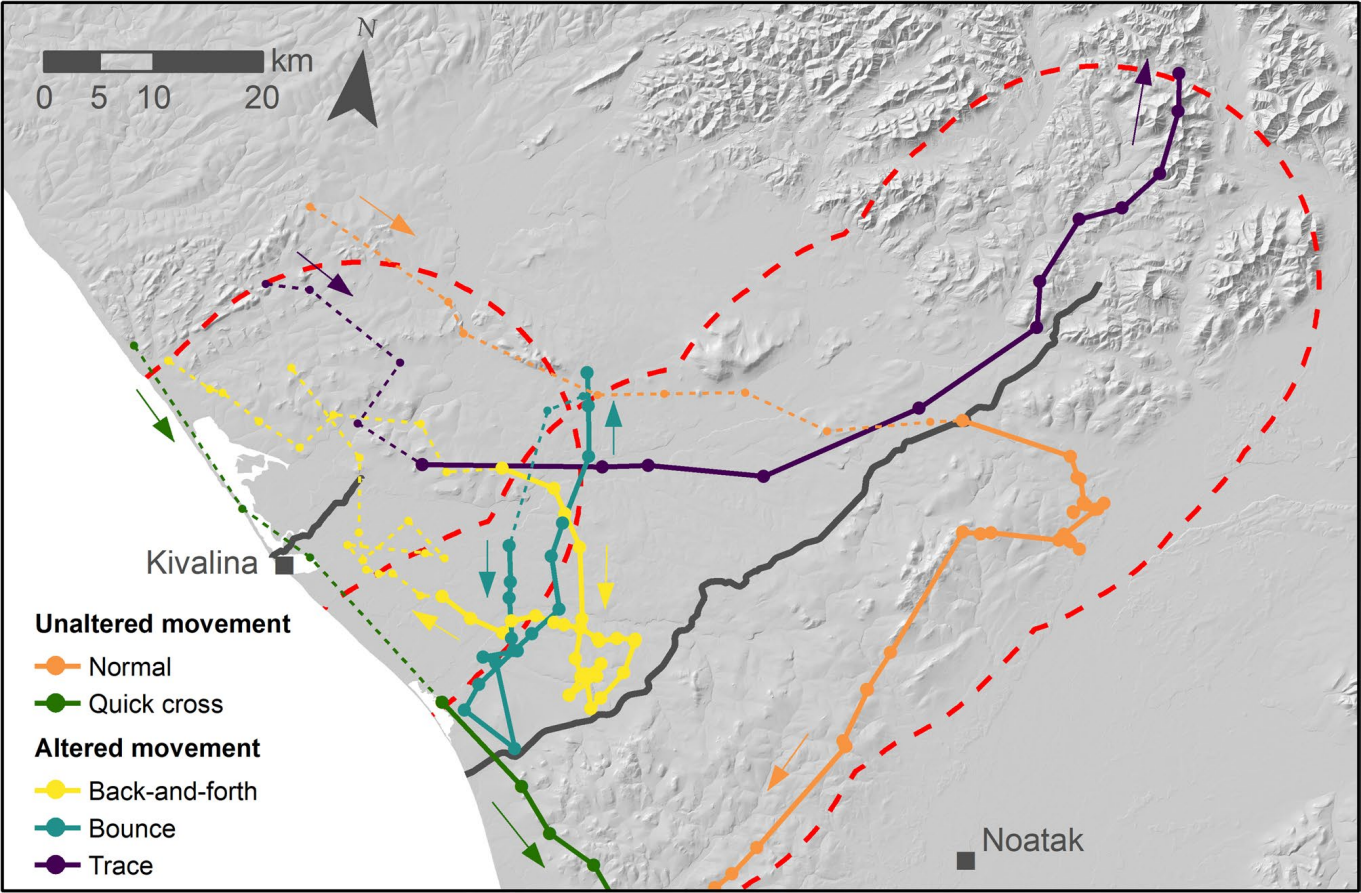
Examples of the five movement behaviors classified by the Barrier Behavior Analysis for adult female Western Arctic Herd caribou in northwestern Alaska, 2009–2024. Caribou locations are depicted as points for five encounters (when a collared animal enters the 20-km focal road buffer, depicted by the dashed red lines). Unaltered movement consisted of normal and quick cross behaviors while altered movement comprised back-and-forth, bounce, and trace behaviors. For each movement type, the example burst (distinguished by an animal crossing the focal road or approaching a neighboring road) is depicted with a solid line and other bursts within the encounter by dotted lines. Direction of movement is indicated with arrows corresponding to the color of the associated movement behavior.

We adopted a modified terminology of classifications to account for the alterations made to the BaBA (Table 1). We defined an “encounter” as the time from which a caribou entered a focal road buffer (typically 20 km, see details below) until it left that buffer (Fig. 2). An encounter could be split into multiple “bursts,” with bursts distinguished if either (1) a focal road was crossed or (2) in the event of overlapping road buffers, the animal moved from being closer to one focal road to another while remaining within the buffers (Fig. 2). As an example of the latter, the back-and-forth burst in Fig. 2 (solid yellow line) started when the caribou moved closer to the Red Dog road than the Kivalina road and ended, switching to a new burst, where the line transitioned to dotted yellow near the northwest-pointing yellow arrow. This occurred because the animal moved away from the Red Dog road to again be closer to the Kivalina road in the dotted portion. Splitting encounters into bursts allowed identification of multiple behavioral responses to a single focal road or to multiple nearby roads with overlapping buffers.

**Table 1:** Key terms used in our modified Barrier Behavior Analysis (BaBA) approach to movement behavior classification of adult female Western Arctic Herd caribou in northwestern Alaska, 2009–2024. See Supplementary Iinformation 1 for more detailed descriptions of model parameters used to classify movement behavior.

Term	Definition
Focal road	One of the five roads examined in this study: Dalton Highway, Kivalina, Kobuk, Nome, and Red Dog. Note that road buffers overlapped for Kivalina and Red Dog so at the encounter scale these were analyzed together.
Encounter	The time from which a caribou entered a focal road buffer until it left the buffer.
Burst	Subdivisions of an encounter, identified by a focal road being crossed or, in the event of overlapping road buffers (Red Dog – Kivalina only), the animal moving from being closer to one focal road to another while remaining within the buffers. Bursts were used to identify multiple behavioral responses to a single focal road or to multiple nearby roads with overlapping buffers.
Unaltered movement	Movements indistinguishable from season-specific movement in the absence of focal roads or for which crossing did not appear hindered. These consisted of normal movement and quick cross behaviors.
Altered movement	Periods where movement behavior changed near a focal road compared to seasonal average movement parameters outside of road buffers. These consisted of back-and-forth, bounce, and trace behaviors.
Normal movement	Movement in which movement parameters fell within seasonal average parameters or all locations in the burst were closest to the same point on the road, which identified situations where a caribou briefly dipped inside a buffer and then exited.
Quick cross	Rapid, linear movement across one or more focal roads, without apparent hindrance by the road.
Back-and-forth	Clustered movement near a focal road, with repeated changes in movement direction leading to relatively confined space use.
Bounce	Movement in which a caribou approached a focal road and then bounced back away, typically without crossing.
Trace	Movement in which a caribou paralleled a road for a sustained amount of time.

The BaBA assigned a movement behavior to each burst (Fig. S1 in Supplementary Information 1). An animal encountering one or more focal roads could have two broad movement responses, each consisting of multiple specific movement behaviors. “Unaltered movements” reflected movements indistinguishable from what was typical for a given season in the absence of focal roads or for which crossing did not appear hindered. In our study, these consisted of “normal movement” and “quick cross” behaviors (Fig. 2; see Supplementary Information 1 for details of each movement behavior classification). The “normal movement” behavior in our study was referred to as “average movement” by Xu et al.^34^, but we modified the terminology to avoid confusion between the behavioral classification and our analysis approach of comparing movement statistics from each burst with the season-specific average and standard deviation of movements outside of focal road buffers (Supplementary Fig. S1). “Quick cross” behaviors reflected rapid, linear movements across focal roads, without apparent hindrance by the feature. We acknowledge that quick cross behavior could be considered a type of altered movement because the animal may increase its movement speed and thus expend additional energy per unit time, however we followed Xu et al.^34^ in labeling this as unaltered due to the road not conspicuously reducing an animal’s mobility or otherwise acting as a barrier.

In contrast to unaltered movement, “altered movement” in our study reflected behavior states where movement behavior changed in proximity to a focal road compared to the seasonal average movement parameters away from roads (Supplementary Fig. S1). These consisted of three different responses that corresponded to the altered movement states used by Xu et al.^34^: “back-and-forth” movements in which the animal clustered movements on one side of a focal road before moving away or crossing; “bounce” movements wherein the animal approached and then bounced back away from a focal road, typically without crossing; and “trace” movements, where the animal paralleled a focal road for a sustained period of time (Fig. 2; Supplementary Information 1). We did not include the “trapped” behavior used by Xu et al.^34^, due to the much lower density of potential barriers in our study system.

Determining a barrier buffer distance to use in a BaBA application is a critical part of the analysis process, as it defines when a road interaction may be detected^34^. We tested a range of focal road buffer distance values (5, 10, 15, 20, and 30 km) surrounding the 15 km buffer distance used by Wilson et al.^33^ to evaluate WAH movement responses to the Red Dog road. We validated classification accuracy at each buffer distance, as well as utility of our modified BaBA approach compared to the default methods from Xu et al.^34^. As described above, we adapted the behavior categories of Xu et al.^34^ to develop an ethogram of five possible behaviors: normal, quick cross, back-and-forth, bounce, and trace. Four expert reviewers with extensive experience with caribou movement data (TJF, KJ, MDC, and DA Hansen from the Alaska Department of Fish and Game) reviewed example burst data to develop a consistent categorization across the ethogram^84,85^. Working with BaBA test runs on a sample set of 11 caribou chosen to represent a variety of sample sizes and durations, a single expert reviewer (TJF) examined each burst’s classification and assigned classification quality (good, decent, questionable, poor) based on how closely the purported classification conformed to the expert-identified ethogram. These tests indicated that a 20 km buffer yielded the highest proportion of good classifications and the highest proportion (0.90) of overall high-quality (good + decent) classifications (Figs. S2 and S3 in Supplementary Information 2), so we used 20 km as the focal road buffer distance in the full analysis. The exception to this was the Kobuk road, for which we used a 5 km buffer based on preliminary testing indicating significant increases in misclassification at larger buffer distances. Together, focal road buffers covered 5.1% of the WAH annual range.

We applied the validation approach used for identifying buffer distances to the results of the BaBA run on our full dataset. There were too many bursts to manually review and characterize so this was done on a stratified random subset. We randomly selected ten bursts from each movement behavior classification and then reviewed and characterized classification quality for the randomly selected bursts and all other bursts within their parent encounter, as described above. All “unknown” behavior classifications (Supplementary Fig. S1) were manually reviewed and assigned to a behavior class based on alignment with the expert-identified ethogram.

In addition to changes to the terminology, we extensively modified the classification workflow to improve classification accuracy for WAH caribou (see Supplementary Information 1 for details). All classification and analyses were conducted in R (Version 4.3.3.) ^86^, with code available at https://github.com/tfullman/BaBA. Results of the BaBA classification that were used for movement behavior analyses were archived on Dryad and are available at 10.5061/dryad.8sf7m0d1n.

### Movement behavior analyses

An individual caribou could have encounters with multiple roads, or multiple encounters with the same road, each year. Additionally, each encounter could have multiple bursts. The potential for multiple bursts and movement behaviors per encounter led us to report two scales of movement responses for WAH caribou: encounter and altered burst scales. At the encounter scale, all bursts within an encounter were combined into a single representation of an interaction with a road or set of roads. Behavior was reduced to a binary of unaltered or altered movement. If any of the bursts within the encounter featured altered movement (back-and-forth, bounce, trace) then the encounter was considered altered. At the altered burst scale, we used characteristics of the altered bursts to identify the specific behavioral responses when altered movement occurred.

At each scale, we reported overall and yearly summary statistics (*n*, min, max, median, and mean) for behavioral responses, seasons, and roads. Season dates followed Joly and Cameron^71^ (spring migration: 1 Apr − 27 May; calving: 28 May − 14 Jun; insect relief: 15 Jun − 14 Jul; late summer: 15 Jul − 31 Aug; fall migration: 1 Sep − 30 Nov; winter: 1 Dec − 31 Mar). Bursts were assigned to the season which occupied the majority of the burst locations. Encounters were assigned to the season of the first burst in the encounter. At the encounter scale, we combined the Red Dog and Kivalina road responses as their overlapping buffers often led to joint reactions in proximity to both roads within a single encounter. At the altered burst scale, each road was distinguished. In addition to counts of encounters and altered bursts, we compared the duration of encounters and altered bursts and quantified road crossing events. We used two-sample Wilcoxon (Mann-Whitney) tests to evaluate differences in duration by season and road^87^ and Pearson’s chi-squared tests for equality of proportions to compare crossing rates^88^.

## Results

The final dataset included caribou locations from 366 animals with sample sizes ranging from 364 to 10,451 locations per individual (median = 2,382; mean = 2,840; sd = 1877). This resulted in a total of 1,039,448 locations that spanned almost 15 years (7 Sep 2009–10 Jul 2024). Running the data through the modified BaBA classification yielded 927 encounters with a focal road (i.e., when a caribou came within the buffer distance of at least one road) that were subdivided into 1,367 bursts. The stratified random validation approach indicated overall strong classification accuracy based on 195 bursts from 85 encounters, with 88.7% of the evaluated bursts receiving “good” or “decent” classifications (Fig. S4 in Supplementary Information 2). There were 49 bursts classified as “unknown” by the BaBA (3.6% of all bursts). Due to missing data, one burst could not confidently be assigned a classification, but we manually reviewed the rest and assigned them to a movement category (31 normal, 5 back-and-forth, 10 bounce, 2 trace).

Overall, 231 caribou (63.1% of collared animals in our dataset) had at least one encounter with a focal road. Of the caribou with at least one encounter, 142 (61.5%) displayed altered movement (had at least one burst with altered movement behavior across their encounters). Some of the caribou had up to 9 altered bursts (median = 1; mean = 1.3; sd = 1.6; Table S1 in Supplementary Information 2). The percentage of caribou with altered movements varied by road, from 8.7% (Kobuk road) to 83.3% (Dalton Highway; Table S2 in Supplementary Information 2). Overall, caribou had 1–16 unique encounters during the study period (median = 3; mean = 4.0; sd = 2.9; Fig. S5 in Supplementary Information 2), consisting of 1–23 total bursts per caribou across all encounters (median = 5; mean = 5.9; sd = 5.0; Fig. S6 in Supplementary Information 2).

**Fig. 3:**
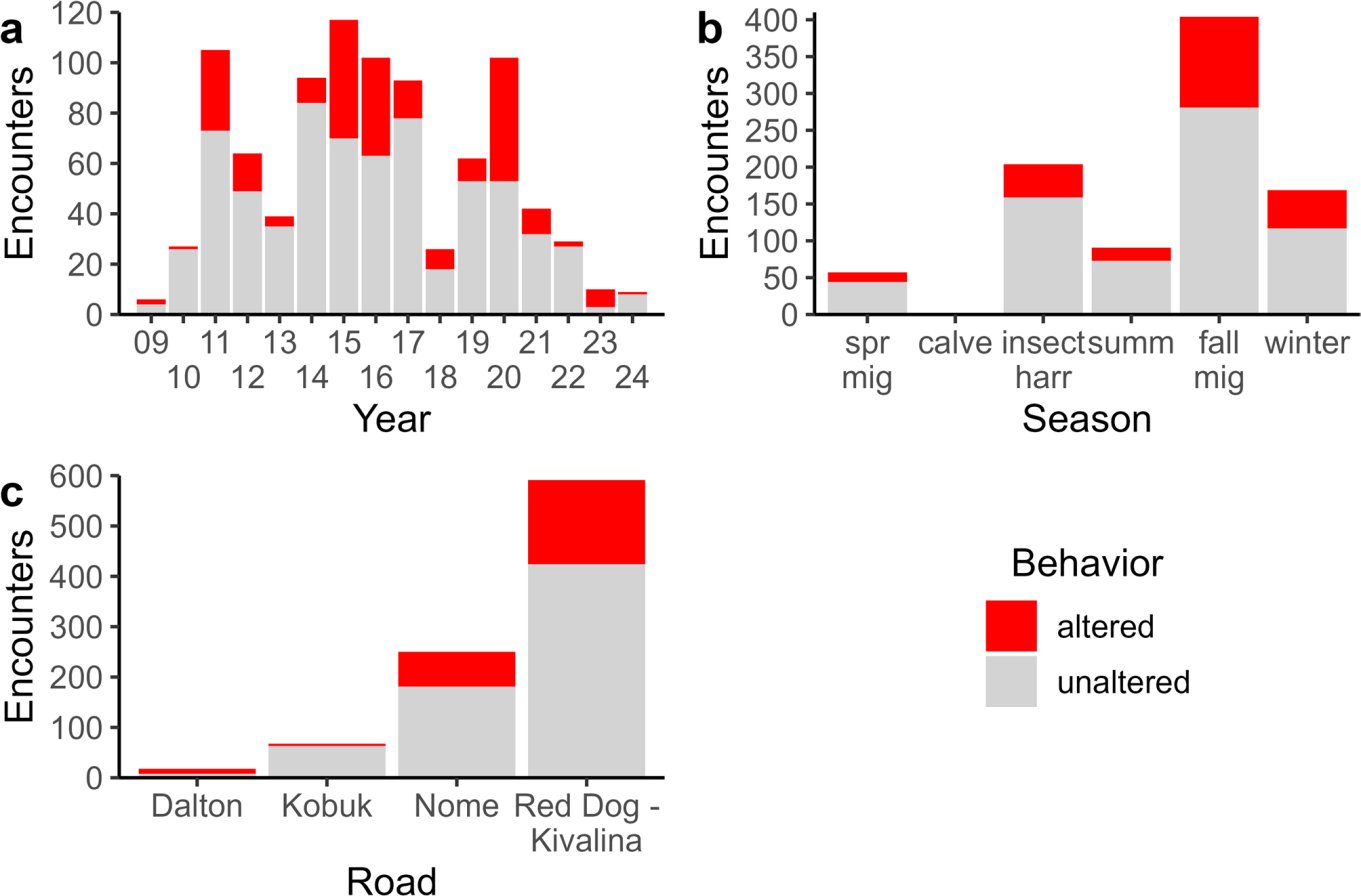
Encounter-scale behavioral responses of adult female Western Arctic Herd caribou to focal roads in northwestern Alaska, 2009–2024. Observed encounters are identified across (**a**) years, (**b**) seasons, and (**c**) roads. Note that y-axes vary between panels. In panel **b** the bar for calving is difficult to see as only two encounters were observed, with no altered movement. spr mig = spring migration, calve = calving, insect harr = insect harassment, summ = summer, fall mig = fall migration.

Looking at the encounter scale, encounters occurred in all years of the study and averaged 58 per year (median = 52; sd = 39.1; Fig. 3a; Table S3 in Supplementary Information 2). Most encounters (79.1%) comprised a single burst, but some included up to 13 bursts (median = 1; mean = 1.5; sd = 1.2; Fig. S7 in Supplementary Information 2). Altered movements were identified in 27.1% of encounters (Table S4 in Supplementary Information 2). Of those encounters with altered movements, a single response was by far most common, though some featured up to eight altered bursts (Supplementary Table S4). While some altered encounters were evident in every year of the study, the relative proportion of altered movements varied over time and ranged between 3.7 and 70.0% (Fig. 3a; Supplementary Table S3; Fig. S8 in Supplementary Information 2). Most encounters with roads occurred during fall migration, followed by the insect harassment season and winter (Fig. 3b; Table S5 in Supplementary Information 2). Apart from the calving season, when encounters with roads were very rare, the percentage of altered movements remained generally consistent across seasons, ranging between about 20–30% of seasonal encounters (Supplementary Table S5; Supplementary Fig. S8). Nearly two-thirds of encounters observed in our dataset were with the Red Dog and Kivalina roads, with Nome road encounters the next most common (Fig. 3c; Table S6 in Supplementary Information 2). Encounters with the Dalton Highway were rare but in each year that they occurred over half led to altered movements (Tables S6 & S7 in Supplementary Information 2; Supplementary Fig. S8).

Caribou with altered movements spent longer in the focal road buffer (mean = 14.3 days; sd = 22.4) than those with unaltered movements (4.9 days; sd = 9.87), as evidenced by significant differences in the duration of encounters (W = 132609; *P* < 0.001; Table 2). Duration of encounters also varied across seasons and roads (Table 2). Encounter duration was longest, on average, during spring migration and winter (11.9 and 10.6 days, respectively; sd = 19.9 and 22.1), but median duration was greatest during fall migration (4.7 days; Table 2). Among focal roads, encounters with the Dalton Highway lasted longest (mean = 33.6 days; sd = 47.0), while those with the Kobuk road were shortest (1.8 days; sd = 1.8; Table 2). Out of the 927 encounters, 160 (17.3%) included one or more road crossings (Table S8 in Supplementary Information 2). Of the total number of instances where caribou crossed focal roads, approximately twice as many happened in encounters with unaltered movements (107) compared to altered movements (53; Supplementary Table S8), however the crossing proportions by behavioral response (15.8% of unaltered encounters included crossings versus 21.1% of altered encounters; Supplementary Table S8) were not significantly different (χ^2^ = 3.22; *P* = 0.073). For animals that crossed, those with altered movements had longer durations of encounters (mean = 20.6 days; sd = 19.8) than caribou with unaltered movements (8.5 days; sd = 16.1; W = 5176; *P* < 0.001; Table 2).

**Table 2:** Duration (days) and number of encounters per variable (*n*) for encounters with focal roads by adult female Western Arctic Herd caribou in northwestern Alaska, 2009–2024.

Grouping	Variable	*n*	min	max	median	mean
Overall		927	0.7	224.7	3.0	7.5
Behavior	Altered	251	0.7	224.7	8.3	14.3
	Unaltered	676	0.7	105.0	2.3	4.9
Season	Spring migration	59	0.7	105.0	3.3	11.9
	Calving	2	2.3	2.7	2.5	2.5
	Insect harassment	204	0.7	17.0	2.3	3.3
	Late summer	91	0.7	12.3	3.0	4.0
	Fall migration	404	0.7	224.7	4.7	8.4
	Winter	167	0.7	130.7	1.7	10.6
Road	Dalton	18	1.0	130.7	5.8	33.6
	Kobuk	68	0.7	10.0	1.3	1.8
	Nome	250	0.7	120.7	3.0	6.7
	Red Dog - Kivalina	591	0.7	224.7	3.3	7.6
Did not cross	Altered	198	0.7	224.7	6.0	12.6
	Unaltered	569	0.7	93.0	2.0	4.2
Crossed road	Altered	53	6.0	120.7	15.7	20.6
	Unaltered	107	0.7	105.0	5.3	8.5

**Fig. 4:**
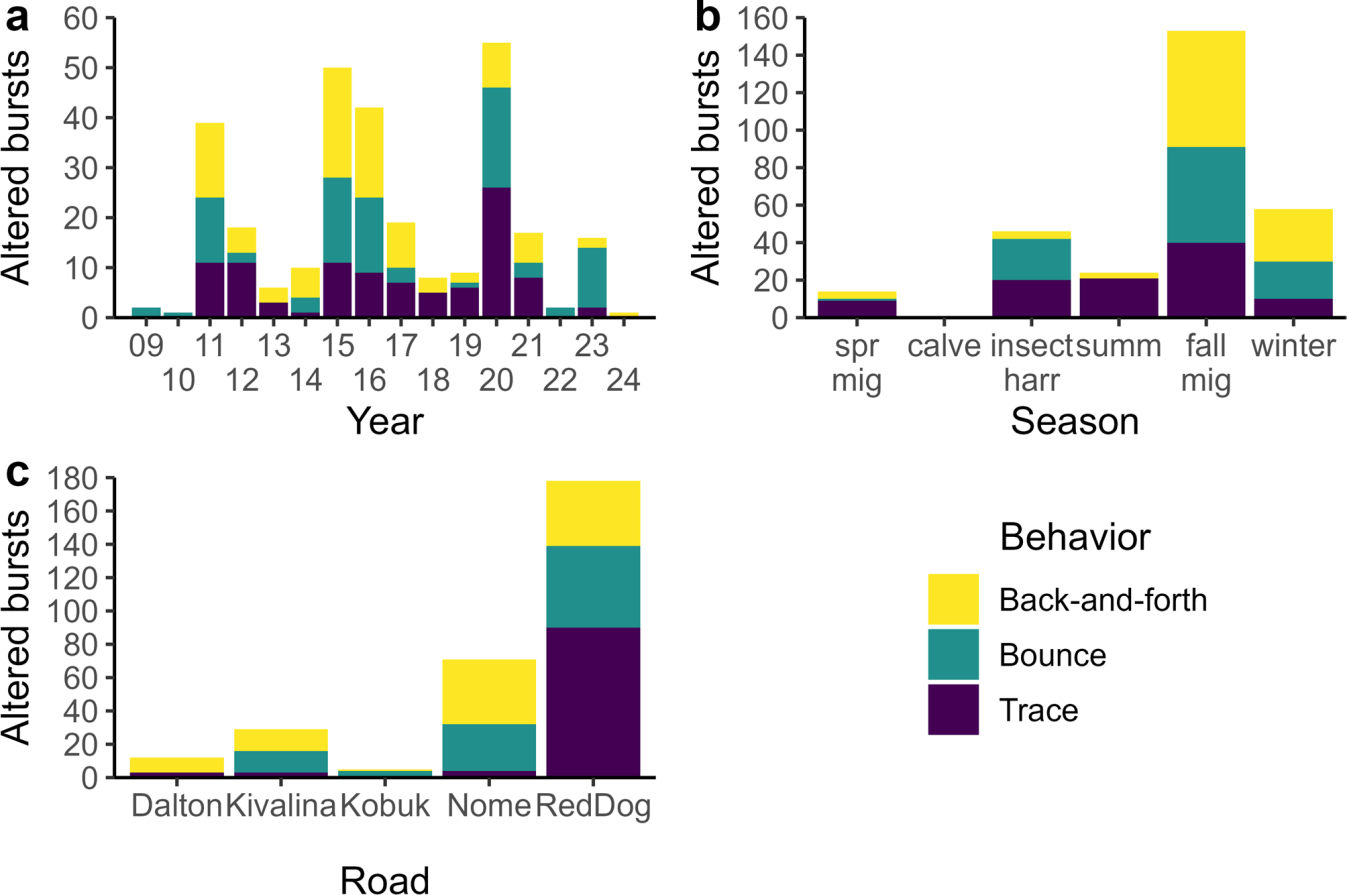
Altered behavioral responses (altered burst scale) of adult female Western Arctic Herd caribou to roads in northwestern Alaska, 2009–2024. The number of altered bursts for each altered behavior class is identified across (**a**) years, (**b**) seasons, and (**c**) roads. Note that y-axes vary between panels. spr mig = spring migration, calve = calving, insect harr = insect harassment, summ = summer, fall mig = fall migration.

Looking at the altered burst scale, there were a total of 295 bursts with altered movements in our dataset. Altered bursts were evenly distributed across the altered movement classes, with 101 back-and-forth behaviors, 94 bounce behaviors, and 100 trace behaviors (Table S9 in Supplementary Information 2). As with responses observed at the encounter scale, the distribution of altered bursts across the three movement classes varied across years (Fig. 4a; Supplementary Table S9). Slightly over half of all altered bursts (51.9%) occurred during fall migration (Fig. 4b; Table S10 in Supplementary Information 2). Most of the altered bursts occurred in proximity to the Red Dog road (60.3%), with about half consisting of trace behavior (Fig. 4c; Table S11 in Supplementary Information 2). In contrast, altered bursts in proximity to the Kivalina road comprised 9.8% of the altered movement responses in the dataset. The Nome road had the second most altered bursts (24.1%), with over half consisting of back-and-forth movements (Supplementary Table S11).

Back-and-forth bursts had the longest average duration (16.8 days; sd = 21.6) of the altered movement types, followed by trace (8.7 days; sd = 9.7), with bounce movements being shortest (3.1 days; sd = 2.1; W_bnf−b_ = 8699, W_bnf−t_ = 7073, W_b−t_ = 1432; all *P* < 0.001; Table S12 in Supplementary Information 2). Altered bursts were longest during winter (mean = 17.2 days, sd = 28.5), followed by spring and fall migration (mean = 10.7 and 9.1 days, respectively; sd = 14.0 and 8.6; Supplementary Table S12). Although interactions with the Dalton Highway were rare (4.1% of all altered bursts; Supplementary Table S11), they exhibited the longest duration of altered movements (mean = 38.7 days; sd = 48.2; Supplementary Table S12). Except for the Kobuk road, which had few altered bursts (1.7% of all altered bursts; Supplementary Table S11) and short durations (mean = 1.5 days; sd = 1.0; Supplementary Table S12), the other roads had similar magnitudes of duration for altered behaviors, ranging between 6.5 and 10.1 days, on average (Supplementary Table S12). Crossing rates for altered movements were generally much lower, at 6.1% (Table S13 in Supplementary Information 2) compared to 17.3% overall for encounters (Supplementary Table S8). This suggests that crossings tended to occur before or after an altered burst (i.e., during unaltered bursts within an encounter), rather than during a burst of altered movement.

## Discussion

Understanding how environmental features, both natural and anthropogenic, facilitate or hinder animal movement is a key aspect of movement ecology^89^ and may suggest management approaches to maintain connectivity for migratory species^32^. Using our modified BaBA approach, we identified and quantified caribou movement responses to roads in northwestern Alaska. While this is among the least developed regions of North America, nearly two-thirds of the collared WAH caribou encountered the five focal roads we analyzed. We found that the focal roads acted as semi-permeable barriers to movement: of the caribou that encountered one or more roads, over 60% displayed altered movement behavior, including bounces away from roads, moving back-and-forth before roads, and tracing along roads. This resulted in more time spent in proximity to roads by caribou with altered movements than by caribou whose behavior was unaltered. Such reactions were not, however, evenly distributed over space and time but tended to occur most often for the Red Dog and Kivalina roads during fall migration.

Our findings align with previous work that identified altered movement behavior for the WAH near the Red Dog road during fall migration^33^. That study used location data from 2009 to 2013 and found that animals with altered movement behavior delayed crossing the road by about 30 days on average^33^. Looking across a longer time period and using a different methodology to detect various types of altered movement behavior, we found altered movements near the Red Dog road have continued. Indeed, across all roads the modified BaBA indicated altered movement behavior in each year of the study, though the proportion of altered events varied over time. Due to the different methodologies employed, we were not able to make direct comparisons with the findings of Wilson et al.^33^, however there was general consistency between our findings and theirs. The Wilson et al.^33^ study found that 28.6% of individuals that came within 15 km of the Red Dog road exhibited delayed crossing, taking 30.2 days longer, on average, than normal crossers. While we did not report time to crossing, we did find that caribou whose movements were altered spent 2–3 times longer in their encounters, regardless of whether they ultimately crossed a road. Our results, in combination with previous road impact studies^31,33,90,91^, suggest a robust indication of road impacts on caribou movement and point to avenues for future study. These include distinguishing the features of roads that lead to barrier effects for caribou (e.g., physical footprint, noise, traffic movement, scents, etc.) and assessing potential energetic, physiological, and survival impacts of altered movements related to roads.

Our findings demonstrate the value of expanding road impact analyses for the WAH beyond the Red Dog road. While much attention has been paid to impacts of mines and associated roads on caribou in Alaska and Canada^33,91–94^, our findings indicate that movement behavior may be altered around other roads as well. Indeed, despite caribou encountering the road relatively far from the town of Nome and it only being travelled by people periodically, the Nome road accounted for nearly a quarter of all altered movement behavior observed in our dataset. Other studies looking at broad-scale habitat use have indicated avoidance of roads by the WAH^95,96^, but this is the first detailed study of WAH responses to roads other than Red Dog. While there may be some relationship between caribou response and the size of the road or traffic level^31,97^, our findings suggest roads of widely varying lengths, designs, and intensities of use can affect movement behavior of caribou. Further research to understand the mechanistic drivers of caribou movement and disturbances by these various roads is a next step in identifying mitigation measures to reduce impacts to caribou movement and facilitate habitat connectivity.

Our findings affirm that most WAH encounters and altered movements occur during fall migration but also identify altered behavior at other seasons of the year, especially during winter and insect harassment. Winter is a critical time for caribou as foraging opportunities are limited and caribou rely heavily on body stores of fat and protein for survival and gestation^98–100^. Further energetic costs at such a time may lead to loss of body mass and depletion of vital energy reserves^101^. Indeed, studies in other ungulate species of displacement and altered habitat use due to energy development have noted that fitness costs are likely greater during winter, when individuals already exhibit a negative energy balance^102^. The impacts of winter disturbance on caribou may be greater in years of severe winter conditions such as high snow depth^101^, when energetic costs of movement increase^103^, foraging opportunities are reduced^101,104,105^, and body mass, protein, and fat loss increase, raising the likelihood of death or reproductive failure^68,99,106–108^. Considering our finding that altered movement bursts lasted the longest during winter, additional research is needed on winter effects of roads on caribou, especially as it intersects with other wintertime environmental conditions.

All collared individuals in our study are adult females, which may limit the inference that can be drawn about responses of WAH juveniles and adult males to focal roads in northwestern Alaska. While movement behavior often differs between male and female ungulates^109–112^ and has been noted for caribou^113,114^, male and female mixing is often high during the fall, especially during the rut^113,114^, which could lead to similar behavior. Furthermore, even though sexual segregation may occur in the distribution of caribou, Cameron & Whitten^113^ found that movement patterns were generally consistent. Whether road permeability is affected by sex differences across seasons is an area for additional study to provide a more comprehensive understanding of behavioral responses to roads by ungulates.

While numerous avenues for additional research remain, our study demonstrates the utility of our modified BaBA for better understanding the nuanced responses to infrastructure and associated anthropogenic activity by migratory ungulates. While other applications of the BaBA and similar approaches have focused on effects of fences^27,34,115–118^, we demonstrate that the tool can be applied to road effects. The BaBA approach identifies multiple movement responses of animals to potential barriers, enabling evaluation of how animal movement is affected by a potential barrier, based on species and season-specific movement data. It also provides multiple quantified metrics like encounter duration, crossing success, and other data that can offer a more nuanced view of how animals interact with potential barriers. Availability of this information may increase opportunities for additional analyses that seek to understand behavioral responses to barriers and their implications for animal movement and fitness^27,34^.

Despite the many benefits of the BaBA technique, one area for future refinement is with barrier buffer identification. Unlike studies that produce a data-driven estimate of the “zone of influence” describing the distance at which a road or other barrier affects animal movement^69,91,93,94^, the BaBA relies on a user-input barrier buffer parameter. We attempted to reduce the arbitrary nature of this buffer distance by testing multiple road buffer distances and selecting the distance at which classification quality was maximized (Supplementary Figs. S2 and S3). Notably, this resulted in the use of a buffer distance (20 km) of similar magnitude to the 16–17 km zone of influence estimate derived for migrating caribou response to industrial roads by Boulanger et al.^91^ and to the 15 km road influence distance used for the WAH by Wilson et al.^33^. Other studies have found variable zone of influence distances across years and seasons for caribou^69,90,94^. Development of more robust means of identifying and validating barrier buffer distances in the BaBA will strengthen the utility of the tool and warrants further research. In addition, while the BaBA identifies behavioral responses to roads, including estimating crossing locations, it does not yield a quantitative estimate of barrier permeability^23^. Such estimates may provide important complementary information that could be combined with BaBA results to more thoroughly compare effects between various potential barriers. We hope that our removal of temporal thresholds in the BaBA workflow and replacement with angle-based movement metrics will expand the utility of the BaBA for other species and study systems and that they can be built upon as described above.

We found that the focal roads in our study present a semi-permeable barrier to caribou movement by quantifying altered movement behavior across years, identifying multiple behavioral responses to roads, and expanding the suite of seasons and roads across which altered movement behavior has been identified. This information can help inform management decisions and potential mitigation measures seeking to balance responsible development with conservation of natural systems and the species and people that rely upon them. Our results also add greater detail and nuance to inform approaches that seek to proactively examine the potential effects of proposed development in the context of infrastructure planning, design, and environmental impact analysis^81,96,119^. Continuing to develop tools like the BaBA will expand the range of contexts in which anthropogenic impacts on animal behavior can be investigated and better understood. Given trends toward expanding infrastructure development^120,121^ and the global challenges facing migratory species^11,122,123^, it is all the more important that new analytical techniques be brought to bear across a wide range of contexts to better inform conservation and management of migratory animals.

## Electronic supplementary material

Below is the link to the electronic supplementary material.

Supplementary Material 1

Supplementary Material 2

## Data Availability

The dataset generated and analyzed in this study is available via the Dryad repository at 10.5061/dryad.8sf7m0d1n.
