# Supplementary material for: Behavioral responses of migratory caribou to semi-permeable roads in Arctic Alaska
Source: Sci Rep. 2025 Jul 9;15:24712. doi: 10.1038/s41598-025-10216-6 (PMC12241334; doi:10.1038/s41598-025-10216-6)
Supplement: Supplementary file 1 — Supplementary Material 1 [file 41598_2025_10216_MOESM1_ESM.pdf]

Fullman, T. J., Joly, K., Gustine, D. D., & Cameron, M. D. 2025. Behavioral responses of migratory caribou to semi-permeable roads in Arctic Alaska. Scientific Reports.
DOI: 10.1038/s41598-025-10216-6.

Supplementary Information 1:
Barrier Behavior Analysis Modifications

We made extensive modifications of the Barrier Behavior Analysis (BaBA) approach developed by Xu et al.[1] to better align with the behavior of Western Arctic Herd (WAH) adult female caribou and improve classification accuracy. The full workflow of our updated BaBA approach is contained in Fig. S1 and can be compared against the BaBA workflow of Xu et al.[1], visualized in their Appendix S1 Figure S1.1. Below we summarize the major alterations between the two approaches, then give detailed descriptions of each behavior class and how it was identified. For a complete accounting of differences between our approach and that of Xu et al.[1], please compare the R code for our modified BaBA approach (https://github.com/tfullman/BaBA) with that of the Xu et al.[1] BaBA approach (https://github.com/wx-ecology/BaBA). To install the latest development version of our modified BaBA package, open an R session and run:

```
devtools::install_github('tfullman/BaBA').
```

Modifications to the BaBA approach

One major change to our BaBA code was altering the workflow to always exclude animal locations within barrier buffers when calculating average and standard deviation of movement parameters for distinguishing normal movement (referred to as "average movement by Xu et al.[1]) from other movement behaviors. This was an option in the Xu et al.[1] approach but was not employed in their study due to the high density of potential barriers in their study system. Roads

in our study occurred at a much lower density so we estimated movement parameters outside of barrier buffers. We also employed season-specific calculations of average and standard deviation of movement parameters, rather than a user-specific moving window. Caribou movement behavior varies widely across seasons[2] and we wanted to ensure that seasonal variation in movement responses did not hinder identification of any alteration of movement behavior due to potential barriers.

Another major change was dropping the user-specified time thresholds for short-duration and long-duration events employed by Xu et al.[1] to instead rely on movement parameters and duration to distinguish between different movement responses. This included an expansion of the movement parameters considered in our BaBA analysis to include movement step turning angles. This allowed us to identify behaviors such as "bounce" based not just on their duration but also on changes in direction and consistency of movement angles in a burst. In our analysis, bounce behavior required an animal to approach and then move away from a potential barrier, while trace behavior required a certain proportion of movements that were roughly parallel to the nearest potential barrier for multiple movement steps (details below).

We followed Hering et al.[3,4] in distinguishing a movement behavior from road crossing. While the definitions of back-and-forth and trace behavior used by Xu et al.[1] included a check for barrier crossings, we allowed barrier crossings in our classifications. Thus, movement behaviors such as back-and-forth or trace sometimes involved a crossing and other times did not. In contrast, the definitions of quick cross and bounce movements did include a check for road crossings.

Rather than using the Xu et al.[1] maximum number of acceptable crossings threshold to account for times when the straight line between subsequent locations might falsely indicate that

a crossing occurred, our BaBA code explicitly distinguished between true and false crossings by testing for locations on either side of a potential barrier. If all locations were on the same side of a potential barrier, we assumed this indicated a false crossing even if the straight line linking subsequent locations intersected the barrier. While we use the terms “true” and “false” crossing for convenience, we cannot know where an animal went between recorded telemetry locations. This is an inherent limitation with any telemetry study. As a consequence, it is possible that even when all observations occurred on one side of a road the animal actually crossed the road and then crossed back within our 8-hour observations. Nonetheless, given the data we have available we assume no crossing occurred if all locations were on the same side of a road.

Movement behavior classifications

We adapted five of the behavior classes identified by Xu et al.[1] for use with WAH caribou. These are described below along with details of the parameters used to classify each behavior (Fig. S1).

Normal movement

Normal movement represented bursts of movement in which movement parameters fell within season average parameters. Specifically, normal movement was classified when the straightness of the movement (str_i, i.e., degree of tortuosity of movement steps within a burst) fell within the season-specific average straightness confidence interval ($str_{mean} - str_{sd} = str_{lower}$, $str_{mean} - str_{sd} = str_{upper}$). In our study, all confidence intervals were defined as the mean ± standard deviation of the given movement statistic. The BaBA contained an `sd_multiplier` parameter that, as its name implies, was multiplied against the standard

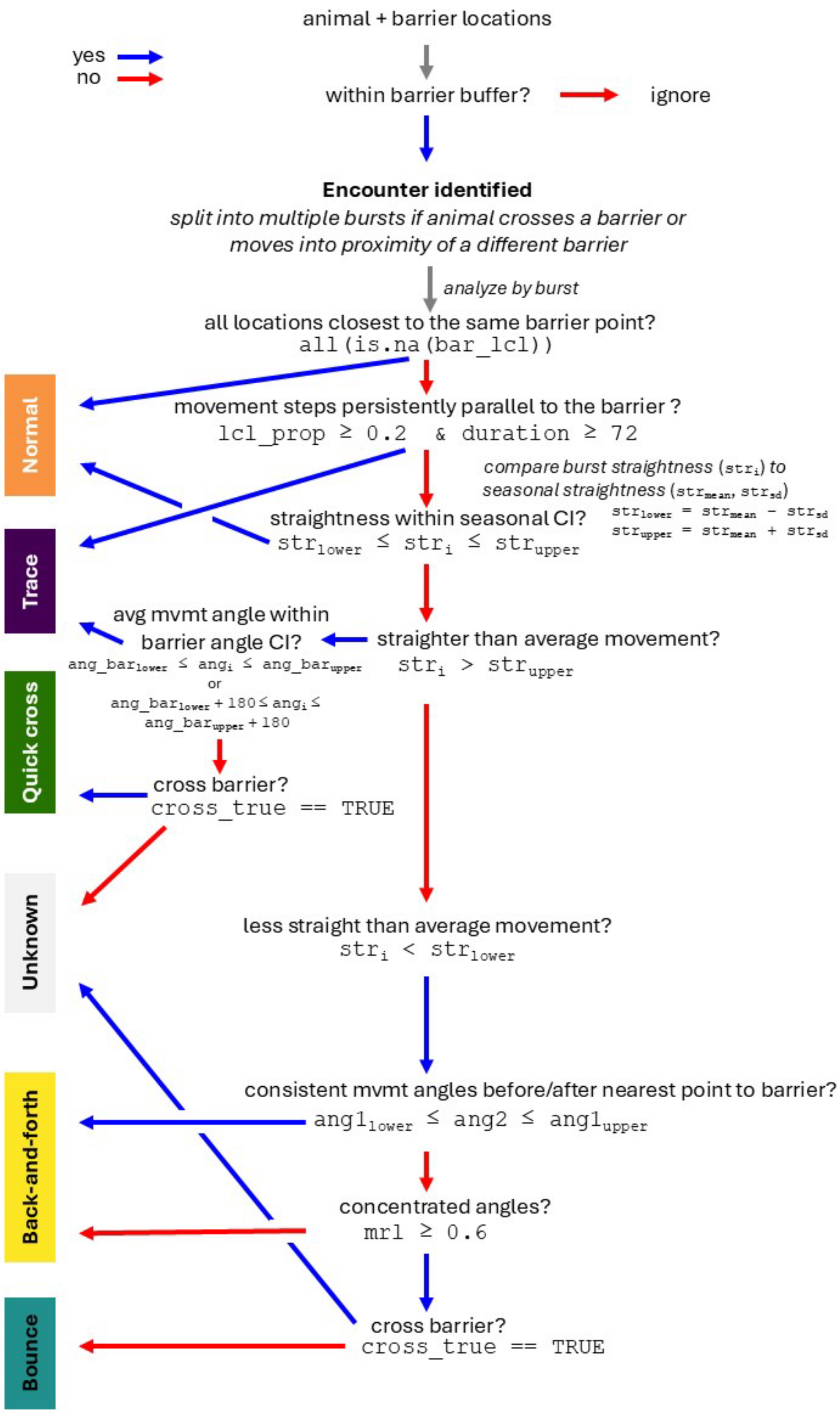

Fig. S1. Classification workflow for the updated Barrier Behavior Analysis, tailored to adult female Western Arctic Herd caribou in northwestern Alaska, 2009–2024. See text for details and parameters used in the classification workflow.

deviation to restrict or relax the range of values considered “normal movement”[1]. We followed Xu et al.[1] in leaving this set to 1 for our analysis. Thus, bursts were classified as normal behavior if $str_{lower} \le str_i \le str_{upper}$ (Fig. S1).

Movement bursts could also be classified as normal if they represented a brief step into a buffer far from the road. This avoided situations where an animal briefly dipped inside a buffer and then exited again rapidly. Such interactions were identified if all locations in the burst were closest to the same point on the road (`all(is.na(bar_lcl))`; Fig. S1).

Quick cross

Quick cross movement was rapid, linear travel across a focal road, without apparent hindrance by the road. To be classified as quick cross, the movement burst had to be 1) straight, 2) roughly perpendicular to the road, and 3) identified as crossing the road (Fig. S1). Specifically, the average movement straightness of the burst had to be straighter than the seasonal average ($str_i > str_{upper}$). The direction of movement also had to be generally perpendicular to the road orientation, lying outside of the road direction confidence interval ($ang_i < ang\_bar_{lower}$ or $ang_i > ang\_bar_{upper}$ or $ang_i < ang\_bar_{lower} + 180$ or $ang_i > ang\_bar_{upper} + 180$). The angle of movement (ang_i) reflected the mean movement angle for the burst. We used circular statistics to calculate all angular mean and standard deviation metrics using the *circular* R package[5]. The angles of each segment of the road line were used to identify the mean and standard deviation of the road angles (`ang_bar`), and thus the confidence interval for road orientation. Note that the addition of 180 to the road orientation confidence interval was included because animal movement is directional (i.e., from the start location of a movement step to the end location) but a barrier direction is not (i.e., roads in this study are traveled in both

directions and could equally be considered as having a heading from right-to-left as left-to-right, depending on which end of the road is chosen as the “start”). For example, if a road arbitrarily is considered to have a 90° orientation, a caribou traveling with a 270° heading would still be traveling parallel to the road, even though it moved in the “opposite” direction of the road orientation. Finally, to be considered quick cross a road crossing had to be indicated. This occurred when caribou locations occurred on either side of a road and the straight line between those locations intersected the road line (`cross_true == TRUE`).

Trace

Trace movements occurred when an animal paralleled a road for a sustained amount of time. Such behavior was identified in two ways. First, for each movement step within a burst we compared the angular heading of the movement step to the angle of the nearest road segments. The start and end points of the movement step were matched with the nearest road points and all intervening road segments were used to calculate the mean and standard deviation of the road angles (`ang_seg`). If the movement angle lay within the road segment confidence interval ($\texttt{ang\_seg}_{lower} \le \texttt{ang}_{i} \le \texttt{ang\_seg}_{upper}$ or $\texttt{ang\_seg}_{lower} + 180 \le \texttt{ang}_{i} \le \texttt{ang\_seg}_{upper} + 180$), then that movement step was considered parallel to the road and we set $\texttt{bar\_lcl}_{i}$ to 1. We calculated the proportion of steps within a burst that were parallel to the road ($\texttt{lcl\_prop} = \texttt{sum(bar\_lcl) / nrow(burst}_{i}\texttt{))}$ and used a threshold of $\texttt{lcl\_prop} \ge 0.2$ to identify trace behavior, based on preliminary testing (Fig. S1). Because trace behavior needs to be persistent, it was only assigned if the duration of the encounter was at least 72 hours ($\texttt{duration} \ge 72$). Second, if the average movement straightness of the burst was straighter than the seasonal average ($\texttt{str}_{i} > \texttt{str}_{upper}$) and the direction of movement was generally parallel to the road

orientation, lying within the road orientation confidence interval ($ang\_bar_{lower} \leq ang_i \leq ang\_bar_{upper}$ or $ang\_bar_{lower} + 180 \leq ang_i \leq ang\_bar_{upper} + 180$) then the BaBA classified it as trace behavior.

Bounce

Bounce movements occurred when an animal approached and then moved away from a focal road, typically without crossing the road. To be classified as a bounce, a movement burst had to feature movements that were less straight than average ($str_i < str_{lower}$; Fig. S1). Bounce movements were concentrated toward the road during the approach and away from the road after bouncing. We split movements before and after the nearest point to the road into two groups and evaluated whether the angular mean of the second group fell outside the confidence interval of the first group, indicating a change in movement heading ($ang2 < ang1_{lower}$ or $ang2 > ang1_{upper}$). We identified the concentration of movements using the mean resultant length, which measured the concentration of data points around a circle[6]. In our case, this reflected the concentration of movement heading angles. Values of mean resultant length range between 0–1 with 0 indicating a large spread and 1 indicating that all data were concentrated in a single point[7]. Based on preliminary testing, we identified bounce movements as having an average mean resultant length value of greater than or equal to 0.6 ($mrl \geq 0.6$).

Back-and-forth

Back-and-forth movements featured repeated changes in movement direction, leading to space use in a relatively confined area. Like bounce movements, back-and-forth movements were also less straight than average ($str_i < str_{lower}$). Because back-and-forth movements lack

consistent headings, they were identified by bursts in which the mean angle of locations after the nearest point to the road was within the confidence interval of the mean locations before the nearest point ($ang1_{lower} \leq ang2 \leq ang1_{upper}$) or those with diffuse angles identified by an average mean resultant length value less than 0.6 ($mrl < 0.6$; Fig. S1).

References

1. Xu, W., Dejid, N., Herrmann, V., Sawyer, H. & Middleton, A. D. Barrier Behaviour Analysis (BaBA) reveals extensive effects of fencing on wide-ranging ungulates. *J. Appl. Ecol.* **58**, 690–698 (2021).
2. Prichard, A. K., Yokel, D. A., Rea, C. L., Person, B. T. & Parrett, L. S. The effect of frequency of telemetry locations on movement-rate calculations in arctic caribou. *Wildl. Soc. Bull.* **38**, 78–88 (2014).
3. Hering, R. *et al.* Don't stop me now: Managed fence gaps could allow migratory ungulates to track dynamic resources and reduce fence related energy loss. *Front. Ecol. Evol.* **10**, 907079 (2022).
4. Hering, R., Hauptfleisch, M., Kramer-Schadt, S., Stiegler, J. & Blaum, N. Effects of fences and fence gaps on the movement behavior of three southern African antelope species. *Front. Conserv. Sci.* **3**, 959423 (2022).
5. Agostinelli, C. & Lund, U. R package 'circular': Circular Statistics. Version 0.5-1. https://CRAN.R-project.org/package=circular (2024).
6. Landler, L., Ruxton, G. D. & Malkemper, E. P. Circular data in biology: advice for effectively implementing statistical procedures. *Behav Ecol Sociobiol* **72**, 128 (2018).

7. Cremers, J. & Klugkist, I. One Direction? A tutorial for circular data analysis using R with examples in cognitive psychology. *Front. Psychol.* **9**, 2040 (2018).
