# Supplementary material for: Behavioral responses of migratory caribou to semi-permeable roads in Arctic Alaska
Source: Sci Rep. 2025 Jul 9;15:24712. doi: 10.1038/s41598-025-10216-6 (PMC12241334; doi:10.1038/s41598-025-10216-6)
Supplement: Supplementary file 2 — Supplementary Material 2 [file 41598_2025_10216_MOESM2_ESM.pdf]

Fullman, T. J., Joly, K., Gustine, D. D., & Cameron, M. D. 2025. Behavioral responses of migratory caribou to semi-permeable roads in Arctic Alaska. Scientific Reports.
DOI: 10.1038/s41598-025-10216-6.

Supplementary Information 2:
Additional Tables and Figures

Table S1. Instances of altered movement bursts per caribou (i.e., number of altered bursts across all encounters for a given caribou), as classified by the modified Barrier Behavior Analysis (BaBA) for adult female Western Arctic Herd caribou in northwestern Alaska, 2009–2024. Only animals with at least one encounter are included.

**altered bursts**	**0**	**1**	**2**	**3**	**4**	**5**	**6**	**7**	**8**	**9**
n caribou	89	70	34	18	8	7	1	3	0	1
%	38.5%	30.3%	14.7%	7.8%	3.5%	3.0%	0.4%	1.3%	--	0.4%

Table S2. Number of caribou encountering each road, and number and percentage of caribou for which movement ever was altered in the presence of each road for adult female Western Arctic Herd caribou in northwestern Alaska, 2009-2024.

**Road**	***n* caribou**	***n* altered**	***n* unaltered**	**% altered**
Dalton	6	5	1	83.3%
Kobuk	46	4	42	8.7%
Nome	84	45	39	53.6%
Red Dog – Kivalina	191	104	87	54.5%

Table S3. Encounter-scale summary across years, indicating the total number of encounters (times when a collared animal entered a focal road buffer) per year and the number with altered and unaltered movements in each year for adult female Western Arctic Herd caribou in northwestern Alaska, 2009–2024. The two percentage columns have different interpretations as the "% total" column indicates the percentage of the total encounters occurring in that particular year, while the "% altered" column indicates the percentage of the encounters in the given year that were altered. For example, 11.3% of all observed encounters occurred in 2011 (% total = 11.3%) and of those, 30.5% showed altered movements (% altered = 30.5%). For results crossing year by road, see Table S7.

**Year**	***n* total**	**% total**	***n* altered**	***n* unaltered**	**% altered**
2009 [a]	6	0.6%	2	4	33.3%
2010	27	2.9%	1	26	3.7%
2011	105	11.3%	32	73	30.5%
2012	64	6.9%	15	49	23.4%
2013	39	4.2%	4	35	10.3%
2014	94	10.1%	10	84	10.6%
2015	117	12.6%	47	70	40.2%
2016	102	11.0%	39	63	38.2%
2017	93	10.0%	15	78	16.1%
2018	26	2.8%	8	18	30.8%
2019	62	6.7%	9	53	14.5%
2020	102	11.0%	49	53	48.0%
2021	42	4.5%	10	32	23.8%
2022	29	3.1%	2	27	6.9%
2023	10	1.1%	7	3	70.0%
2024 [a]	9	1.0%	1	8	11.1%
Total	927		251	676	

[a] Note that 2009 and 2024 are incomplete years as 2009 records begin in September and 2024 records end in July.

Table S4. Instances of altered movement (mvmt) bursts per encounter with a focal road (encounter scale) classified by the modified Barrier Behavior Analysis (BaBA) for adult female Western Arctic Herd caribou in northwestern Alaska, 2009–2024.

***n* altered mvmt**	**0**	**1**	**2**	**3**	**4**	**5**	**6**	**7**	**8**
n encounters	676	222	21	5	2	0	0	0	1
%	72.9%	23.9%	2.3%	0.5%	0.2%	--	--	--	0.1%

Table S5. Encounter-scale summary across seasons, indicating the total number of encounters per season and the number with altered and unaltered movements in each season for adult female Western Arctic Herd caribou in northwestern Alaska, 2009–2024. The two percentage columns have different interpretations as the "% total" column indicates the percentage of the total encounters occurring in a particular season, while the "% altered" column indicates the percentage of the encounters in the given season that included altered movement. For example, 43.6% of all observed encounters occurred during fall migration and, of those, 30.4% showed altered movements.

**Season**	***n* total**	**% total**	***n* altered**	***n* unaltered**	**% altered**
Spring migration	57	6.1%	13	44	22.8%
Calving	2	0.2%	0	2	0.0%
Insect harassment	204	22.0%	45	159	22.1%
Late summer	91	9.8%	18	73	19.8%
Fall migration	404	43.6%	123	281	30.4%
Winter	169	18.2%	52	117	30.8%

Table S6. Encounter-scale summary across focal roads for adult female Western Arctic Herd caribou in northwestern Alaska, 2009–2024, indicating the total number of encounters per road and the number with altered and unaltered movements for each road. At the encounter scale, overlapping buffers for the Red Dog and Kivalina roads often led to interactions with both roads so they are shown combined. The two percentage columns have different interpretations as the "% total" column indicates the percentage of the total encounters occurring for a particular road, while the "% altered" column indicates the percentage of the encounters with the given road that included altered movement. For example, 27.0% of all observed encounters occurred with the Nome road and, of those, 27.6% showed altered movements. For results crossing year by road, see Table S7.

**Road**	***n* total**	**% total**	***n* altered**	***n* unaltered**	**% altered**
Dalton	18	1.9%	10	8	55.6%
Kobuk	68	7.3%	5	63	7.4%
Nome	250	27.0%	69	181	27.6%
Red Dog – Kivalina	591	63.8%	167	424	28.3%

Table S7. Percentage of encounters with altered movements by year and road for adult female Western Arctic Herd caribou in northwestern Alaska, 2009–2024. Blank values indicate no encounters were recorded for that year–road combination.

Year	Dalton	Kobuk	Nome	Red Dog – Kivalina
2009 [a]			33.3%	
2010		0.0%	20.0%	0.0%
2011	54.5%	12.5%	8.3%	37.1%
2012	57.1%	0.0%	14.3%	20.4%
2013			16.7%	9.1%
2014		0.0%	16.3%	6.2%
2015			34.7%	63.6%
2016			35.6%	41.9%
2017			25.0%	15.7%
2018		0.0%	0.0%	33.3%
2019		0.0%		15.0%
2020		40.0%		48.5%
2021		0.0%		43.5%
2022		15.4%		0.0%
2023				70.0%
2024 [a]		0.0%		16.7%

[a] Note that 2009 and 2024 are incomplete years as 2009 records begin in September and 2024 records end in July.

Table S8. Encounter-scale crossing data for adult female Western Arctic Herd caribou in northwestern Alaska, 2009–2024. The "*n* encounters" column indicates the total number of encounters in each grouping. The "*n* cross" column indicates the number of encounters for a given grouping that had one or more crossings.

Grouping	Variable	*n* encounters	*n* cross	% cross
Overall		927	160	17.3%
Behavior	Altered	251	53	21.1%
	Unaltered	676	107	15.8%
Season	Spring migration	59	9	15.3%
	Calving	2	1	50.0%
	Insect harassment	204	34	16.7%
	Late summer	91	0	0.0%
	Fall migration	404	105	26.0%
	Winter	167	11	6.6%
Road	Dalton	18	2	11.1%
	Kobuk	68	14	20.6%
	Nome	250	12	4.8%
	Red Dog - Kivalina	591	132	22.3%

Table S9. Altered burst-scale summary across years for adult female Western Arctic Herd caribou in northwestern Alaska, 2009–2024, indicating the total number of bursts with altered movement per year and how these are divided across the three altered movement behaviors. The total percentage column indicates the percentage of the total altered movement bursts occurring in a particular year, while the behavior-specific percentage columns indicate the percentage of altered bursts in the given year falling under the specified movement classification. For example, 18 altered bursts were reported in 2012 (6.1% of all altered bursts). Of those, 5 were back-and-forth movements (27.8% of the altered bursts in that year), 2 were bounce (11.1%), and 11 were trace (61.1%).

	Total		Back-and-forth		Bounce		Trace	
**Year**	***n***	**%**	***n***	**%**	***n***	**%**	***n***	**%**
2009 *	2	0.7%	0	--	2	100.0%	0	--
2010	1	0.3%	0	--	1	100.0%	-	--
2011	39	13.2%	15	38.5%	13	33.3%	11	28.2%
2012	18	6.1%	5	27.8%	2	11.1%	11	61.1%
2013	6	2.0%	3	50.0%	0	--	3	50.0%
2014	10	3.4%	6	60.0%	3	30.0%	1	10.0%
2015	50	16.9%	22	44.0%	17	34.0%	11	22.0%
2016	42	14.2%	18	42.9%	15	35.7%	9	21.4%
2017	19	6.4%	9	47.4%	3	15.8%	7	36.8%
2018	8	2.7%	3	37.5%	0	--	5	62.5%
2019	9	3.1%	2	22.2%	1	11.1%	6	66.7%
2020	55	18.6%	9	16.4%	20	36.4%	26	47.3%
2021	17	5.8%	6	35.3%	3	17.6%	8	47.1%
2022	2	0.7%	0	--	2	100.0%	0	--
2023	16	5.4%	2	12.5%	12	75.0%	2	12.5%
2024 *	1	0.3%	1	100.0%	0	--	0	--
Total	295		101		94		100	

* Note that 2009 and 2024 are incomplete years as 2009 records begin in September and 2024 records end in July.

Table S10. Altered burst-scale summary across seasons, indicating the total number of bursts with altered movement per season and how these are divided across the three altered movement behaviors for adult female Western Arctic Herd caribou in northwestern Alaska, 2009–2024. The total percentage column indicates the percentage of the total altered movement bursts occurring in a particular season, while the behavior-specific percentage columns indicate the percentage of altered bursts in the given season falling under the specified movement classification.

	**Total**		**Back-and-forth**		**Bounce**		**Trace**	
**Season**	***n***	**%**	***n***	**%**	***n***	**%**	***n***	**%**
Spring migration	14	4.7%	4	28.6%	1	7.1%	9	64.3%
Calving	0	--	--	--	--	--	--	--
Insect harassment	46	15.6%	4	8.7%	22	47.8%	20	43.5%
Late summer	24	8.1%	3	12.5%	0	--	21	87.5%
Fall migration	153	51.9%	62	40.5%	51	33.3%	40	26.1%
Winter	58	19.7%	28	48.3%	20	34.5%	10	17.2%

Table S11. Altered burst-scale summary across roads, indicating the total number of bursts with altered movement per road and how these are divided across the three altered movement behaviors for adult female Western Arctic Herd caribou in northwestern Alaska, 2009–2024. Unlike at the encounter scale, at the burst level each altered movement could be matched with a particular road so Red Dog and Kivalina responses are distinguished below. The total percentage column indicates the percentage of the total altered movement bursts occurring for a particular road, while the behavior-specific percentage columns indicate the percentage of altered bursts for the given road falling under the specified movement classification.

	**Total**		**Back-and-forth**		**Bounce**		**Trace**	
**Road**	***n***	**%**	***n***	**%**	***n***	**%**	***n***	**%**
Dalton	12	4.1%	9	75.0%	0	--	3	25.0%
Kivalina	29	9.8%	13	44.8%	13	44.8%	3	10.3%
Kobuk	5	1.7%	1	20.0%	4	80.0%	0	--
Nome	71	24.1%	39	54.9%	28	39.4%	4	5.6%
Red Dog	178	60.3%	39	21.9%	49	27.5%	90	50.6%

Table S12. Duration (days) and number per variable (*n*) for altered bursts of adult female Western Arctic Herd caribou in northwestern Alaska, 2009–2024.

Grouping	Variable	*n*	min	max	median	mean
Overall		295	0.7	130.7	5.7	9.7
Behavior	Back-and-forth	101	1.0	130.7	10.7	16.8
	Bounce	94	0.7	15.0	2.3	3.1
	Trace	100	1.0	81.0	6.3	8.7
Season	Spring migration	14	1.3	39.3	4.2	10.7
	Calving	0	--	--	--	--
	Insect harassment	46	1.3	7.7	2.7	3.4
	Late summer	24	3.0	12.3	8.3	7.2
	Fall migration	153	0.7	81.0	6.7	9.1
	Winter	58	1.0	130.7	7.0	17.2
Road	Dalton	12	2.7	130.7	12.3	38.7
	Kivalina	29	1.3	19.0	5.0	6.5
	Kobuk	5	1.0	3.3	1.0	1.5
	Nome	71	1.0	101.3	6.7	10.1
	Red Dog	178	0.7	81.0	5.7	8.3
Did not cross	Back-and-forth	96	1.0	130.7	10.7	16.1
	Bounce	93	0.7	15.0	2.3	3.1
	Trace	88	1.0	81.0	6.2	9.0
Crossed road	Back-and-forth	5	2.0	101.3	12.7	30.9
	Bounce	1	3.3	3.3	3.3	3.3
	Trace	12	2.7	9.7	8.0	7.0

Table S13. Altered burst-scale crossing data for adult female Western Arctic Herd caribou in northwestern Alaska, 2009–2024. The "*n* bursts" column indicates the total number of altered bursts in each grouping. The "*n* cross" column indicates the number of altered bursts for the given grouping where a crossing occurred.

Grouping	Variable	*n* bursts	*n* cross	% cross
Overall		295	18	6.1%
Behavior	Back-and-forth	101	5	5.0%
	Bounce	94	1	1.1%
	Trace	100	12	12.0%
Season	Spring migration	14	1	7.1%
	Calving	0	--	--
	Insect harassment	46	0	0.0%
	Late summer	24	6	25.0%
	Fall migration	153	5	3.3%
	Winter	58	6	10.3%
Road	Dalton	12	2	16.7%
	Kivalina	29	0	0.0%
	Kobuk	5	0	0.0%
	Nome	71	4	5.6%
	Red Dog	178	12	6.7%

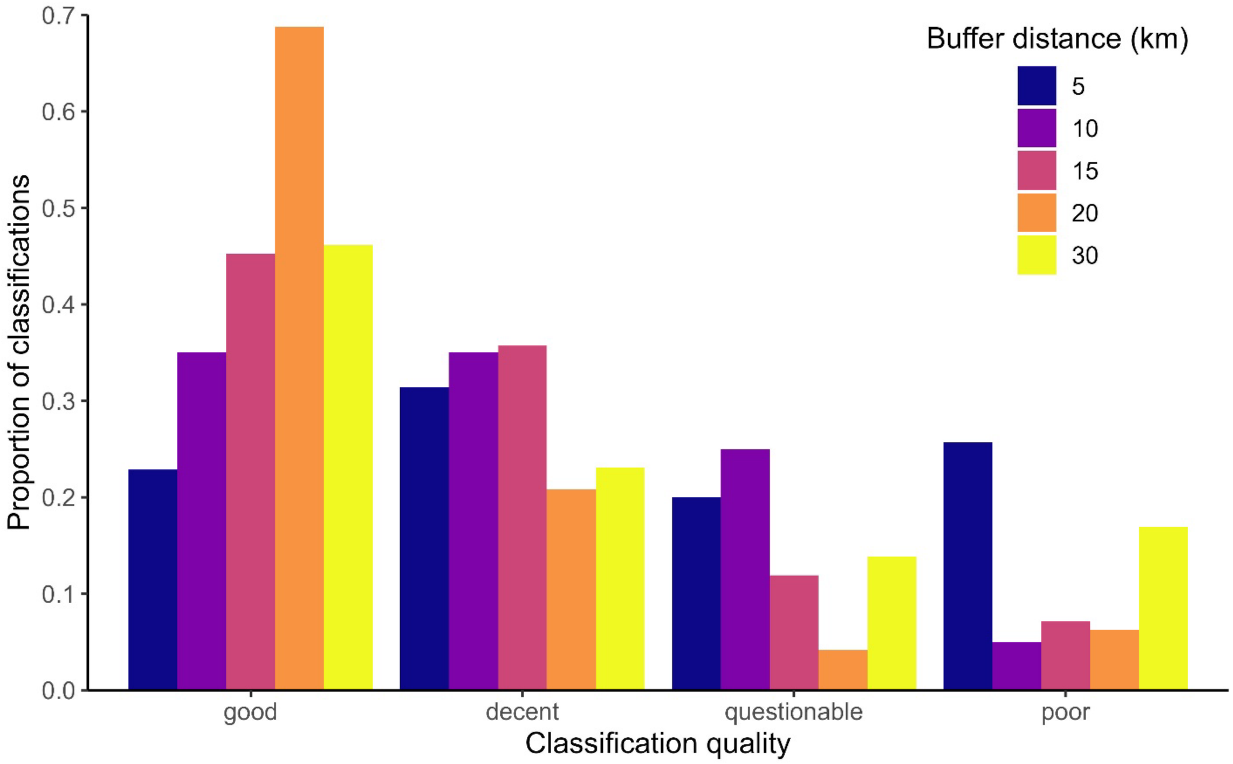

Fig. S2. Classification quality at various barrier road buffer distances, indicated by preliminary testing on a sample set of data for 11 adult female Western Arctic Herd caribou in northwestern Alaska, 2009–2024. For each buffer distance, all identified bursts were manually reviewed and assigned to one of four categories based on classification alignment with a pre-defined ethogram: good, decent, questionable, or poor.

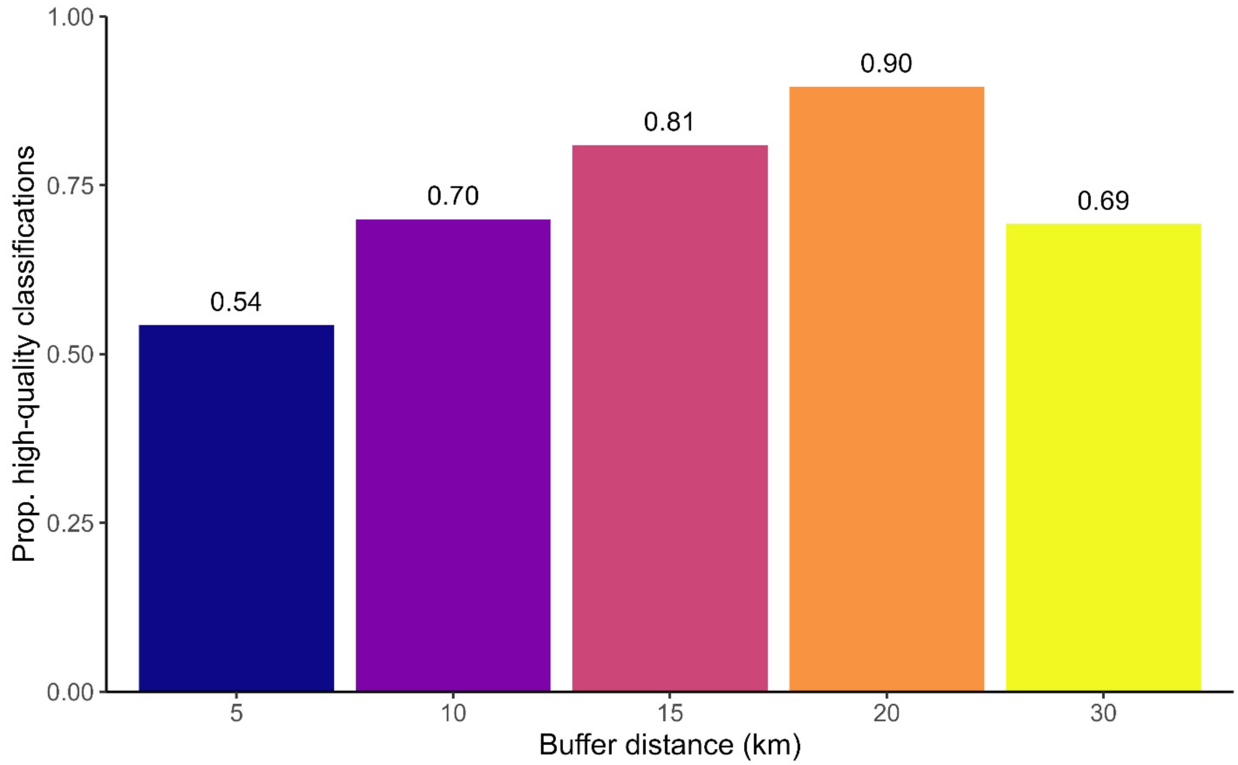

Fig. S3. Proportion of high-quality classifications (good + decent) at various road buffer distances, indicated by preliminary testing on a sample set of data for 11 adult female Western Arctic Herd caribou in northwestern Alaska, 2009–2024.

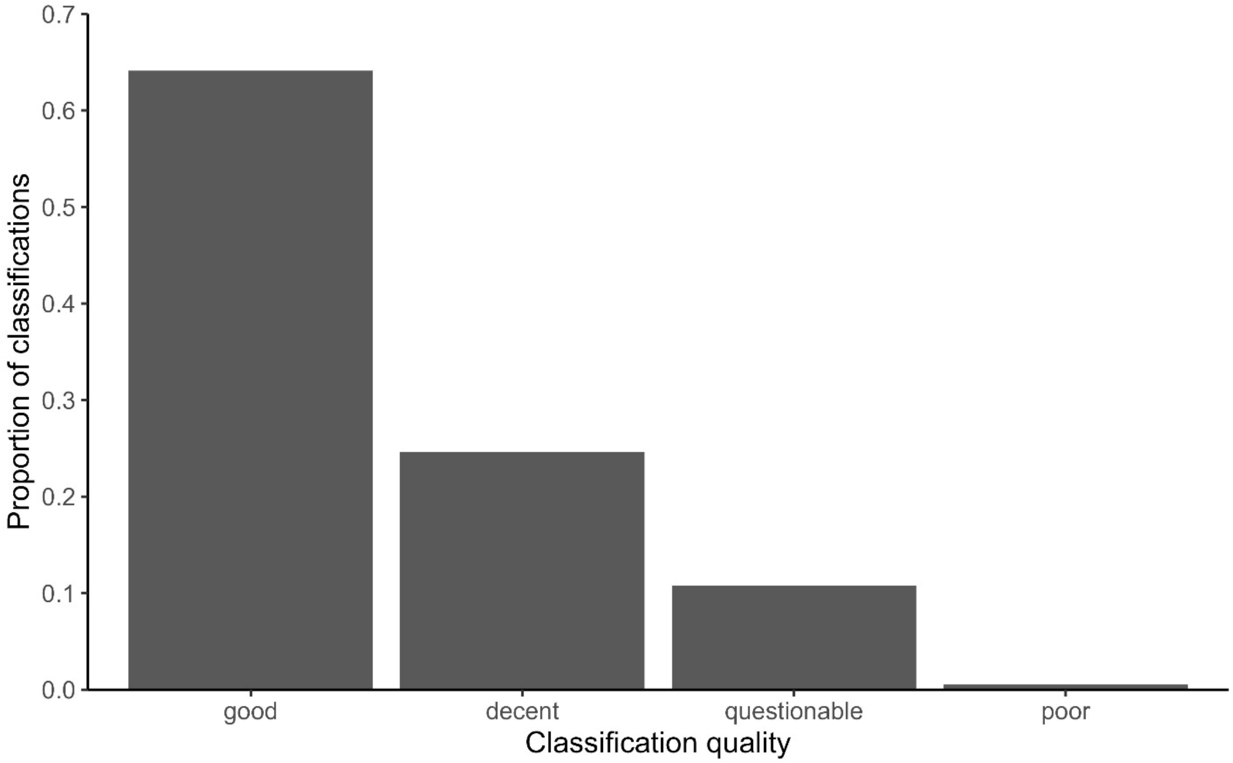

Fig. S4. Proportion of classifications at each quality level for a random stratified sample consisting of 195 bursts (14% of all bursts) from the final dataset for adult female Western Arctic Herd caribou in northwestern Alaska, 2009–2024.

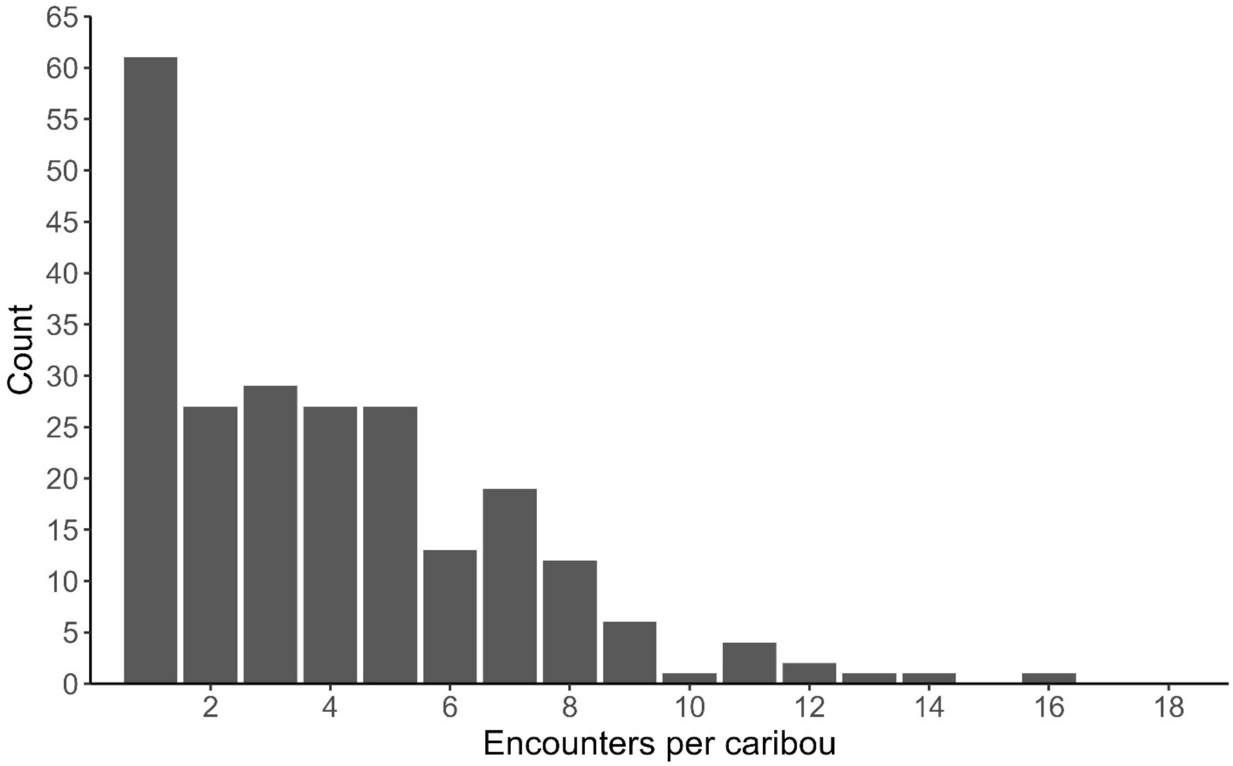

Fig. S5. Observed number of encounters per caribou in our study of adult female Western Arctic Herd caribou in northwestern Alaska, 2009–2024. We defined an "encounter" as one interaction of a caribou with a focal road, consisting of the time from which an animal enters within a focal road buffer until it leaves that buffer. Only caribou that came within 20 km of a focal road (i.e., that had an encounter) were included here. Caribou that encountered at least one focal road exhibited between 1–16 unique encounters (median = 3; mean = 4.0).

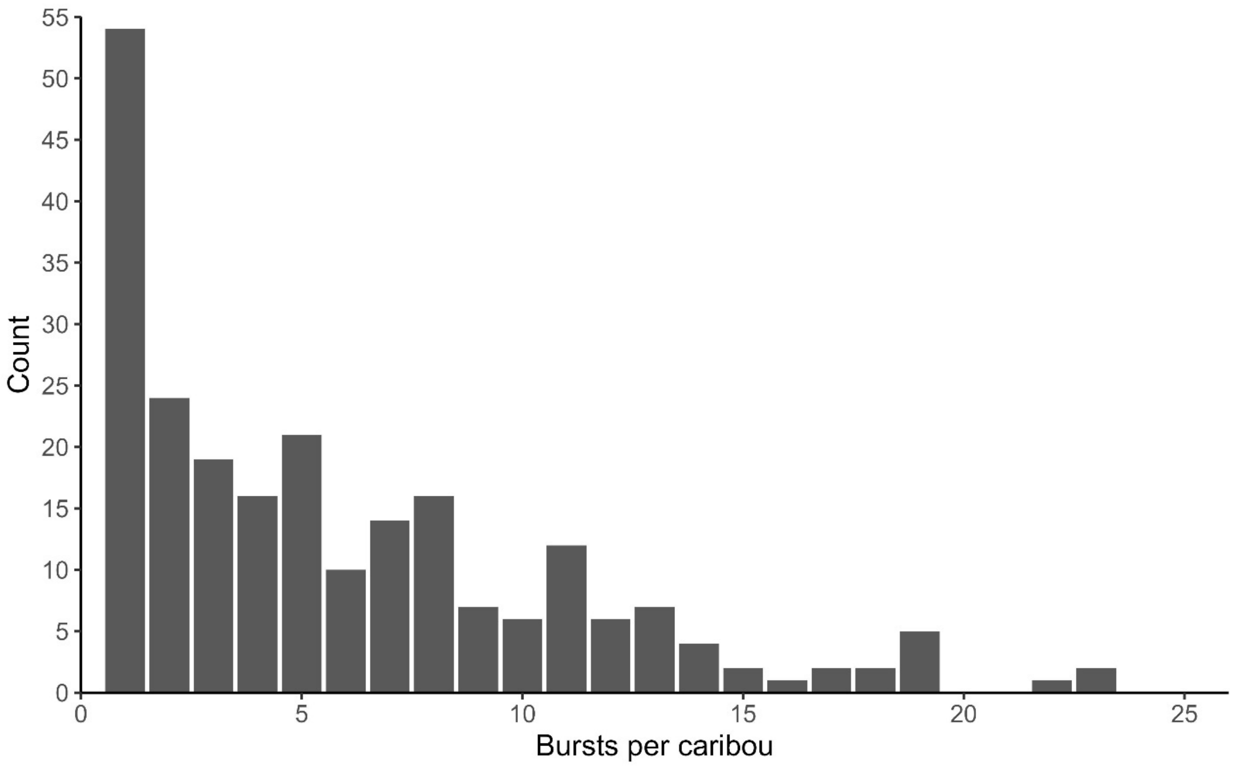

Fig. S6. Observed number of bursts per caribou in our study of adult female Western Arctic Herd caribou in northwestern Alaska, 2009–2024. Each encounter (when the animal entered a focal road buffer) could be split into multiple "bursts," with bursts distinguished if a road was crossed or the animal moved from proximity of one road to another while remaining within the road buffer. Use of bursts allowed identification of multiple behavioral responses to a single road, or to multiple nearby roads with overlapping buffers. Only caribou that came within 20 km of a road (i.e., that had an encounter) were included here. Caribou that encountered at least one road exhibited between 1–23 total bursts (median = 5; mean = 5.9).

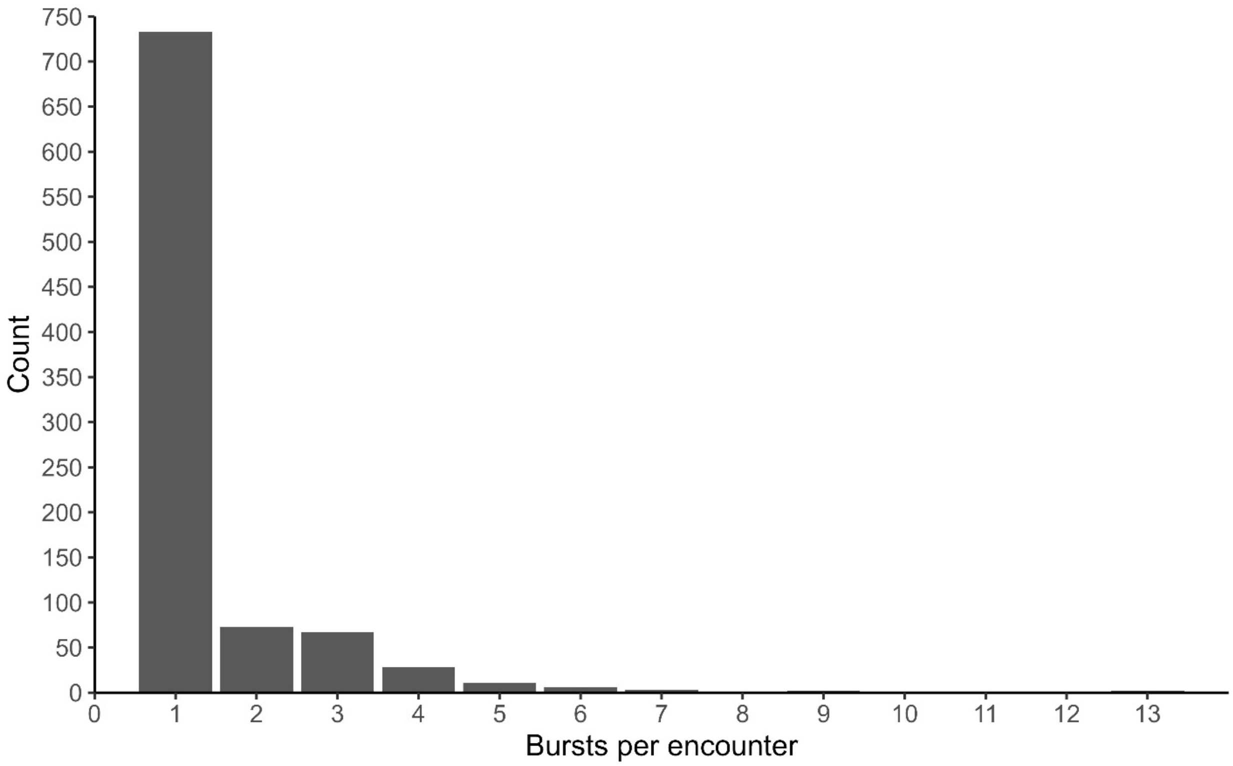

Fig. S7. Observed number of bursts per encounter for adult female Western Arctic Herd caribou in northwestern Alaska, 2009–2024. Encounters comprised between 1–13 bursts (median = 1; mean = 1.5).

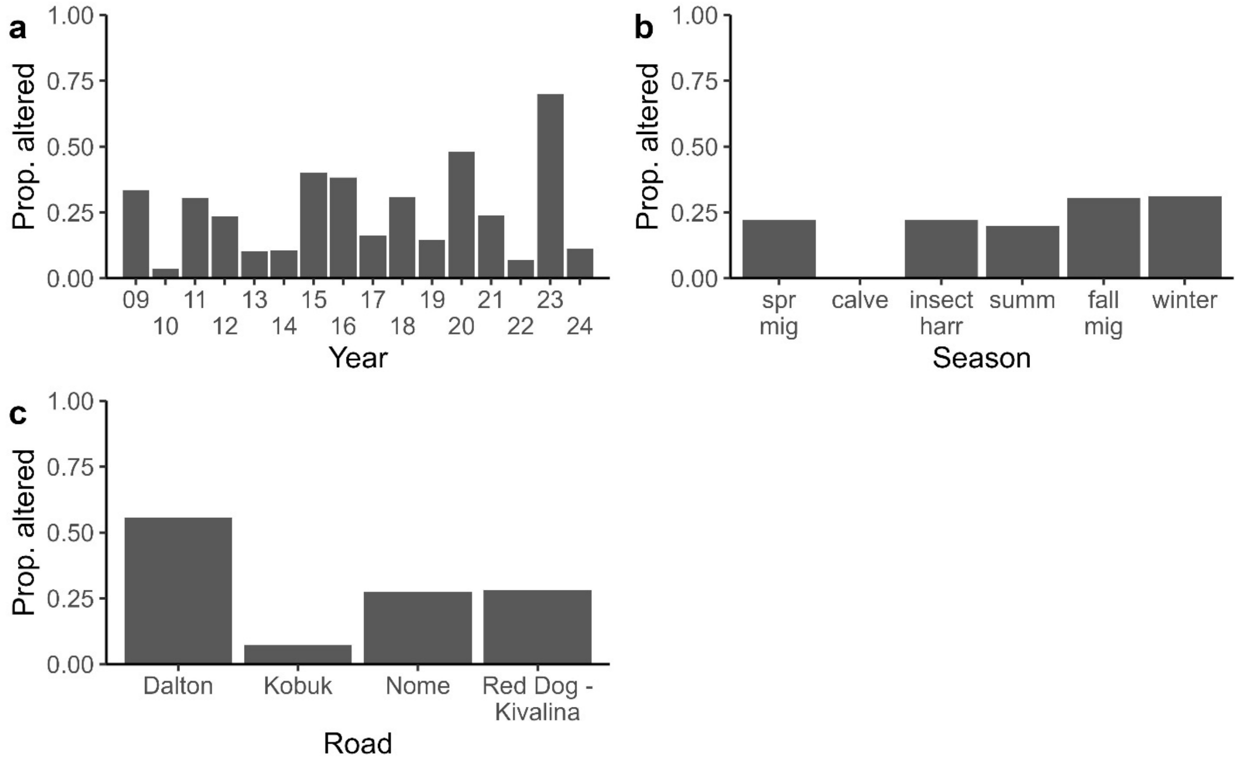

Fig. S8. Encounter-scale behavioral responses of adult female Western Arctic Herd caribou to focal roads in northwestern Alaska, 2009–2024. The proportion of encounters with altered movement behavior is identified across a) years, b) seasons, and c) roads. spr mig = spring migration, calve = calving, insect harr = insect harassment, summ = summer, fall mig = fall migration.
